# Long-term exposure to food-grade disinfectants causes cross-resistance to antibiotics in *Salmonella enterica* serovar Typhimurium strains with different antibiograms and sequence types

**DOI:** 10.1186/s13756-023-01333-w

**Published:** 2023-12-13

**Authors:** Ricardo A. Wu-Chen, Jinsong Feng, Mohamed Elhadidy, Reshma B. Nambiar, Xinyu Liao, Min Yue, Tian Ding

**Affiliations:** 1 Department of Food Science and Nutrition, College of Biosystems Engineering and Food Science, Zhejiang University, Hangzhou, 310058 China; 2 Biomedical Sciences Program, University of Science and Technology, Zewail City of Science and Technology, Giza, Egypt; 3 Center for Genomics, Helmy Institute for Medical Sciences, Zewail City of Science and Technology, Giza, Egypt; 4 Department of Bacteriology, Mycology and Immunology, Faculty of Veterinary Medicine, Mansoura University, Mansoura, Egypt; 5 College of Animal Sciences, Zhejiang University, Hangzhou, 310058 China; 6 Future Food Laboratory, Innovation Center of Yangtze River Delta, Zhejiang University, Jiaxing, 314100 China

**Keywords:** Disinfectant, Antimicrobial resistance, *Salmonella* Typhimurium, Sequence type, ST34

## Abstract

**Background:**

Disinfectants are important in the food industry to prevent the transmission of pathogens. Excessive use of disinfectants may increase the probability of bacteria experiencing long-term exposure and consequently resistance and cross-resistance to antibiotics. This study aims to investigate the cross-resistance of multidrug-resistant, drug-resistant, and drug-susceptible isolates of *Salmonella enterica* serovar Typhimurium (*S*. Typhimurium) with different sequence types (STs) to a group of antibiotics after exposure to different food-grade disinfectants.

**Methods:**

A panel of 27 *S*. Typhimurium strains with different antibiograms and STs were exposed to increasing concentrations of five food-grade disinfectants, including hydrogen peroxide (H_2_O_2_), benzalkonium chloride (BAC), chlorine dioxide (ClO_2_), sodium hypochlorite (NaClO), and ethanol. Recovered evolved strains were analyzed using genomic tools and phenotypic tests. Genetic mutations were screened using breseq pipeline and changes in resistance to antibiotics and to the same disinfectant were determined. The relative fitness of evolved strains was also determined.

**Results:**

Following exposure to disinfectants, 22 out of 135 evolved strains increased their resistance to antibiotics from a group of 14 clinically important antibiotics. The results also showed that 9 out of 135 evolved strains had decreased resistance to some antibiotics. Genetic mutations were found in evolved strains. A total of 77.78% of ST34, 58.33% of ST19, and 66.67% of the other STs strains exhibited changes in antibiotic resistance. BAC was the disinfectant that induced the highest number of strains to cross-resistance to antibiotics. Besides, H_2_O_2_ induced the highest number of strains with decreased resistance to antibiotics.

**Conclusions:**

These findings provide a basis for understanding the effect of disinfectants on the antibiotic resistance of *S*. Typhimurium. This work highlights the link between long-term exposure to disinfectants and the evolution of resistance to antibiotics and provides evidence to promote the regulated use of disinfectants.

**Supplementary Information:**

The online version contains supplementary material available at 10.1186/s13756-023-01333-w.

## Introduction

*Salmonella enterica* is a leading cause of bacterial foodborne illnesses, causing enteritis outbreaks resulting in significant morbidity and even death [1, 2]. *Salmonella enterica* has been widely isolated from food, human, farm animal, and environmental sources [3], making it a pathogen of high concern. Serovar Typhimurium is among the most common serovars causing human salmonellosis outbreaks every year [4], especially those with multilocus sequence types (STs) ST34 and ST19 [5]. In recent decades, the emergence of antibiotic resistance in *Salmonella enterica* serovar Typhimurium (*S*. Typhimurium) has been observed worldwide [5]. The emergence and rapid spread of *S*. Typhimurium ST34, which harbors antibiotic resistance, has caused public health concerns and is associated with both animal and human infections [6]. The emergence of resistance to food-grade disinfectants and even cross-resistance to antibiotics has been observed in *S*. Typhimurium exposed to disinfectant treatment [7, 8].

Disinfectants or biocides are commonly used in many settings, including food processing, animal husbandry, healthcare, and households, to control harmful microorganisms and avoid infections. Sub-inhibitory concentrations of disinfectants can occur frequently due to many factors, such as inadequate dilution ratios, inappropriate storage of formulations, or high amounts of organic matter [8]. Moreover, some disinfectants, such as quaternary ammonium compounds, remain stable for short- and long-term usage [9]. Currently, global policies for antibiotic use are strict. Disinfectants, in turn, are less restricted than antibiotics. Therefore, there is an urgent need to promote the regulated use of disinfectants to curb the emergence of antibiotic resistance promoted by long-term exposure to disinfectants.

Although disinfectants are found everywhere and are used in many daily life activities, the evolution of disinfectant resistance is less studied than that of antibiotics. There is increasing concern that food-grade disinfectants have aggravated antibiotic resistance issues [9]. Previous studies suggest that disinfectant exposure could exert selective pressure that consequently selects for cross-resistance to antibiotics. Exposure of *Pseudomonas aeruginosa* (*P. aeruginosa*) to increasing concentrations of benzalkonium chloride (BAC) has been shown to select for mutations in the *pmrB* gene and overexpression of *mexCD-oprJ* multidrug efflux pump genes that contribute to polymyxin B resistance [9]. In *Acinetobacter baumannii* (*A. baumannii*), a subinhibitory BAC concentration promotes the emergence of mutants with reduced susceptibility to aminoglycoside antibiotics [10]. In *Escherichia coli* (*E. coli*), BAC tolerance was associated with reduced cell surface charge and mutation in the *lpxM* locus [11]. However, the genetic basis of adaptation to different disinfectants and the effect on antibiotic resistance remain unclear.

Therefore, the aim of this study was to determine the cross-resistance of *S.* Typhimurium strains with different antibiograms and STs to a group of antibiotics following exposure to increasing concentrations of disinfectants commonly used in the food industry, including BAC, hydrogen peroxide (H_2_O_2_), chlorine dioxide (ClO_2_), sodium hypochlorite (NaClO), and ethanol.

## Materials and methods

### Bacterial strains and culture conditions

A panel of 27 *S.* Typhimurium clinical isolates from food poisoning were used in this study (Table 1). The panel consisted of 11 multidrug-resistant isolates, 9 drug-resistant isolates, 5 susceptible isolates, and 2 reference strains (ATCC 14028 and D23580) according to AMR gene screening and phenotype prediction by the Staramr pipeline. Strains belonged to different STs, including major STs ST34 and ST19, and minor STs ST36, ST99, and ST313 (Additional file 1) [12]. For data analysis, the 2 reference strains were grouped together with the sensitive group. Unless otherwise specified, strains were cultured in Luria–Bertani (LB) [13] broth at 37 °C with agitation at 175 rpm to produce a final concentration of 10^9^ CFU/mL.

**Table 1:** List of *S*. Typhimurium isolates used in this study

No	Strain number	ST type	Collection date	Location
			**(yyyy-mm-dd)**	
*Multidrug-resistant*				
1	SAL02010	34	2013–08-02	Hangzhou
2	SAL02073	34	2013–08-02	Hangzhou
3	SAL02152	34	2015–10-21	Hangzhou
4	SAL02135	34	2015–06-04	Hangzhou
5	SAL02304	34	2017–11-29	Hangzhou
6	SAL02249	19	2017–05-25	Hangzhou
7	SAL02214	19	2016–09-25	Hangzhou
8	SAL02239	19	2016–12-08	Hangzhou
9	SAL02683	19		Zhejiang
10	SAL02041	34	2013–08-02	Hangzhou
11	SAL02047	19	2013–08-02	Hangzhou
*Drug-resistant*				
12	SAL02000	34	2013–08-02	Hangzhou
13	SAL02003	19	2013–08-02	Hangzhou
14	SAL02005	19	2013–08-02	Hangzhou
15	SAL02006	19	2013–08-02	Hangzhou
16	SAL02008	36	2013–08-02	Hangzhou
17	SAL02219	19	2016–10-21	Hangzhou
18	SAL02030	34	2013–08-02	Hangzhou
29	SAL02069	36	2013–08-02	Hangzhou
20	SAL02203	36	2016–07-27	Hangzhou
*Susceptible*				
21	SAL01711	34		
22	SAL02017	19	2013–08-02	Hangzhou
23	SAL02046	36	2013–08-02	Hangzhou
24	SAL02070	34	2013–08-02	Hangzhou
25	SAL02685	99	2015	Wuhan
*Reference strain*				
26	ATCC 14028	19		
27	D23580	313	2004	Malawi

### Minimum inhibitory concentration (MIC) of antibiotics

The MICs of the 14 different antibiotics listed in Table 2 were determined by broth microdilution according to the Clinical and Laboratory Standards Institute (CLSI) M07 (11th edition) guidelines [14]. A serial dilution of each antibiotic was prepared in the range of 1024–0.125 mg/L. Volumes of 100 μL of serially diluted antibiotics were added to 96-well plates. Isolates were inoculated onto culture plates containing Mueller*–*Hinton agar *(*MHA) and incubated for 24 h at 37 °C. One colony from each isolate from the inoculated plates was inoculated in Mueller–Hinton broth (MHB) and incubated at 37 °C for 3–4 h until the OD_600_ reached 0.1. Afterwards, the culture broth was diluted 10^–3^ times, and 100 μL was added to 96-well plates containing antibiotics to obtain a final bacterial concentration of 5 × 10^5^ CFU/mL. ATCC 25922 and ATCC 27853 strains were used as quality controls. The positive control was 200 μL of inoculum without antibiotic treatment, and the negative control was 100 μL of MHB and 100 μL of diluted antibiotic. The MIC of all antibiotics for all isolates was determined before (wild type) and after (evolved) the laboratory evolutionary experiment. The experiment was repeated at least two times on different days.

**Table 2:** Antibiotics that were screened for MIC in this study. A group of 14 clinically important antibiotics belonging to 9 antibiotic classes were tested for MIC

Antibiotic class	Antibiotic agent	Abbreviation	Breakpoint (μg/mL)^a^		
			S	I	R
β-lactam	Ampicillin	AMP	≤ 8	16	≥ 32
	Amoxicillin-clavulanate	AMC	≤ 8/4	16/8	≥ 32/16
Aminoglycoside	Gentamicin	GEN	≤ 4	8	≥ 16
	Kanamycin	KAN	≤ 16	32	≥ 64
	Streptomycin^b^	STR	≤ 8	16	≥ 32
Tetracycline	Tetracycline	TET	≤ 4	8	≥ 16
Macrolide	Azithromycin	AZI	≤ 16	–	≥ 32
Quinolone	Nalidixic acid	NAL	≤ 16	–	≥ 32
	Ciprofloxacin	CIP	≤ 0.06	0.12–0.5	≥ 1
Phenicol	Chloramphenicol	CHL	≤ 8	16	≥ 32
Folate pathway inhibitor	Trimethoprim-sulfmethaxazole	TST	≤ 2/38	–	≥ 4/76
Cephem	Ceftiofur	CF	≤ 2	4	≥ 8
	Cefoxitin	CX	≤ 8	16	≥ 32
Carbapenem	Imipenem	IMI	≤ 1	2	≥ 4

^a^*S* sensitive, *I* intermediate, *R* resistant

^b^For streptomycin, the same MIC breakpoint for netilmicin was used

### MICs of disinfectant

The MICs of the five disinfectants for all wild-type and evolved strains were determined by broth microdilution (Table 3). BAC, H_2_O_2_, and ethanol were diluted in MHB. NaClO and ClO_2_ were diluted in phosphate buffered saline (PBS) instead of MHB to avoid neutralization by the organic matter in the media. Volumes of 100 μL of serially diluted disinfectants were correspondingly added to 96-well plates. Strains were inoculated and incubated in 96-well plates as described previously. The experiment was repeated at least two times on different days.

**Table 3:** Disinfectants used in this study and their corresponding mechanism of action and MIC values

	Strain	MIC (μg/mL) before evolution				
		Hydrogen peroxide (H_2_O_2_)	Benzalkonium chloride (BAC)	Chlorine dioxide (ClO_2_)	Sodium hypochlorite (NaClO)	Ethanol
	Primary mechanism of action*﻿	Oxidation of thiol groups and disulfide bonds of proteins	Inactivation of energy-producing enzymes, denaturation of essential cell proteins, and disruption of cell membrane	DNA damage and protein denaturation by oxidation	Phospholipid destruction and oxidation	Membrane damage and denaturation of protein
*Multidrug-resistant*						
1	SAL02010	56	11	116	68	100,000
2	SAL02073	64	11	116	98	140,000
3	SAL02152	64	10.5	116	86	130,000
4	SAL02135	64	12	116	86	140,000
5	SAL02304	64	10.5	116	92	140,000
6	SAL02249	64	9.5	116	92	140,000
7	SAL02214	64	11.5	116	92	130,000
8	SAL02239	68	12.5	116	92	140,000
9	SAL02683	64	9.5	116	92	120,000
10	SAL02041	64	11	68	86	140,000
11	SAL02047	64	9	116	92	120,000
*Drug-resistant*						
12	SAL02000	64	9.5	116	92	140,000
13	SAL02003	64	11	116	92	140,000
14	SAL02005	64	10	116	92	120,000
15	SAL02006	64	10	116	92	120,000
16	SAL02008	64	12	116	98	140,000
17	SAL02219	64	10	116	80	120,000
18	SAL02030	64	11.5	116	92	140,000
29	SAL02069	64	11.5	116	92	140,000
20	SAL02203	64	10.5	116	92	140,000
*Susceptible*						
21	SAL01711	60	10.5	116	80	120,000
22	SAL02017	60	9.5	56	86	100,000
23	SAL02046	60	10	116	92	130,000
24	SAL02070	60	11	116	104	140,000
25	SAL02685	58	9	116	56	140,000
*Reference strain*						
26	ATCC 14028	64	10	104	92	100,000
27	D23580	64	12	128	104	140,000

*[41]

### Laboratory evolutionary experiment

Isolates were preliminarily evolved by culturing them in the presence of disinfectants as described previously [15]. Strains were inoculated in 96-well plates following the same procedure as described above. The starting concentration of each of the disinfectants was half MIC (0.5 MIC), and cultures were grown at 37 °C for 72 h. Subcultures were made by transferring 2 μL of the inoculum to a new well, which contained 1.5× the previous concentration of disinfectant at a volume of 198 μL. Strains were grown for several generations until no growth was observed after 72 h.

### Stability of evolved resistance

To test the stability of the evolved resistance to disinfectants, the evolved strains were repeatedly transferred to LB broth without disinfectant every 24 h for 7 consecutive days, and then the MIC of disinfectant and antibiotics was determined.

### DNA extraction and whole-genome sequencing (WGS)

The wild-type and selected evolved strains (evolved strains that showed changes in MICs of antibiotics) following disinfectant exposure were subjected to second-generation whole-genome shotgun sequencing (WGS) to identify mutations linked to disinfectant exposure. Genomic DNA (gDNA) was extracted from 1 mL of culture from each strain using a TIANamp bacteria DNA kit (Tiangen Biotech, Beijing, China) following the manufacturer’s instructions. DNA concentration and purity were measured by Qubit®3.0 Fluorometer (Invitrogen, USA). A total amount of 0.2 μg DNA per sample was used as input material for the DNA library preparations. A sequencing library was generated using the NEB Next® Ultra™ DNA Library Prep Kit for Illumina (NEB, USA) following the manufacturer’s recommendations, and index codes were added to each sample. Briefly, genomic DNA samples were fragmented by sonication to a size of 350 bp. Then, DNA fragments were end polished, A-tailed, and ligated with the full-length adapter for Illumina sequencing, followed by further PCR amplification. After PCR products were purified by the AMPure XP system (Beckman Coulter, Beverly, USA), libraries were analyzed for size distribution by NGS3K/Caliper and quantified by real-time PCR (3 nM). The clustering of the index-coded samples was performed on a cBot Cluster Generation System using a Nextera XT DNA library construction kit according to the manufacturer’s instructions (FC-131-1024; Illumina, US) and sequenced by the Illumina sequencing platform NovaSeq 6000 (Illumina, USA). After cluster generation, the DNA libraries were sequenced on an Illumina platform, and 2 × 150 bp paired-end reads were generated.

### Bioinformatics analysis

Sequence reads were assembled and annotated using the Comprehensive Genome Analysis Service provided by the Bacterial and Viral Bioinformatics Resource Center (BV-BRC 3.25.0) (https://www.bv-brc.org/). We used Unicycler (version 0.4.8) for assembly [16]. A 2-round polishing step was performed using Pilon (version 1.23) to improve the assembly. The minimum contig length was set to 300, and contig coverage was set to 5. The quality of the assembled genomes was assessed by QUAST 5.0.2. Genomes were annotated using the PATRIC RASTtk-enabled genome annotation service [17]. AMR phenotypes were predicted using the Staramr pipeline [12].

Variation calling of each evolved strain was performed with breseq (version 0.35.5) [18] in consensus mode against corresponding wild-type isolates. Variations of H14028 were called against the ATCC14028 reference genome (NCBI RefSeq assembly accession: GCF_003864015.1) and C-D23580 against the D23580 reference genome (NCBI RefSeq assembly accession: GCF_000027025.1).

### Fitness measurements

Overnight cultures of each wild-type and selected evolved strain were adjusted to OD_600_ = 0.1 and then diluted 10^–3^ times. Growth curves were assessed in 96-well plates incubated at 37 °C with shaking at 180 rpm. OD_600_ measurements were performed at 30-min intervals for the first 6 h, 1-h intervals from 6 to 12 h, and 12-h intervals from 12 to 24 h. Fitness (W) was measured as the area under the growth curve recorded in biocide-free medium. The relative fitness of the evolved strains was calculated by dividing the fitness of evolved strains by the fitness of the corresponding parental strain. Relative changes in fitness were tested with a one-sample *t* test for μ = 1 using GraphPad Prism 9.0.0 (GraphPad Software, Boston, MA, USA). A *P* value equal to or less than 0.05 was considered significant.

## Results and discussion

### Evolution in food-grade disinfectants selected for strains with increased resistance to disinfectants

**Fig. 1:**
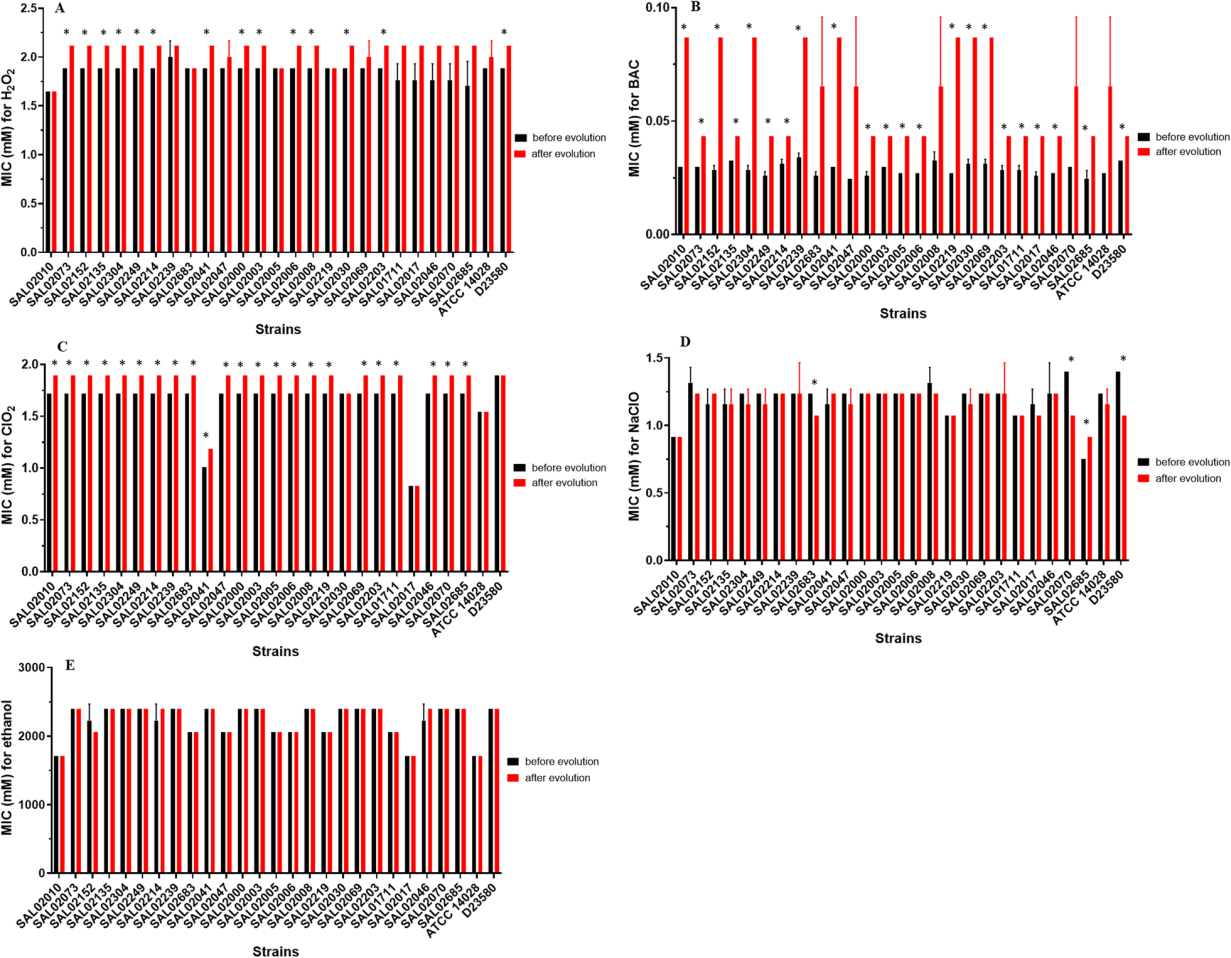
MICs of H_2_O_2_ (**A**), BAC (**B**), ClO_2_ (**C**), NaClO (**D**), and ethanol (**E**) for *S*. Typhimurium strains before and after evolution in the presence of the same disinfectant. Strains were exposed to increasing concentrations of disinfectant for approximately 300–500 generations. MIC was screened before the onset of evolution and after evolution. Significance of difference of evolved to wild type strain indicated by asterisks: *P ≤ 0.05 (one sample *t* test for μ = 1)

The *S*. Typhimurium strains evolved in the presence of several disinfectants commonly used in the food industry for approximately 300–500 generations. There were noticeable differences between the disinfectants in terms of susceptibility decrease to the same disinfectant and the development of cross-resistance to antibiotics. Overall, strains exposed to BAC, H_2_O_2_, and NaClO showed increased direct resistance to the same disinfectant (Fig. 1A–C). Only one strain exposed to ClO_2_ showed increased resistance to ClO_2_ (Fig. 1D), and no strain showed increased resistance to ethanol after exposure to ethanol (Fig. 1E). Strains exposed to BAC showed the highest increase in direct resistance to BAC, while strains exposed to ethanol did not show changes in direct resistance to ethanol.

As shown in Fig. 1B, BAC-evolved strains showed increased resistance to BAC. This resistance was found to persist after 7 consecutive transfers in antimicrobial-free culture media. BAC is a quaternary ammonium compound that is widely used as an active ingredient in many products, including disinfectants, antiseptics, preservatives, eye drops, and shampoo. It has a wide range of applications in household, industrial, healthcare, agriculture and food production settings [11, 19]. Efflux pumps are the main mechanism for increased resistance to BAC [20]. Previous studies have shown that BAC resistance is mediated by mutations in genes that encode efflux pumps or by acquiring efflux pump genes from the gene pool by horizontal gene transfer [11]. In this scenario, agricultural soils, river sediments, and wastewaters are places where bacterial communities may encounter residues of BAC that were applied in the abovementioned settings.

In addition to the low stability of H_2_O_2_ compared to other disinfectants, selection is not uniform and usually depends on the probability of the sublethal concentration and the starting genetic background [21]. Therefore, not all H_2_O_2_-evolved strains showed increased resistance to H_2_O_2,_ including SAL02010, SAL2683, SAL02005, and SAL02219 (Fig. 1A). H_2_O_2_ is an oxidizing agent widely used as a disinfectant. H_2_O_2,_ like other oxidative biocides, oxidizes the electrons removed from susceptible chemical groups and becomes reduced in the process [22]. There is evidence that H_2_O_2_ induces the release of DNA in *Streptococcus gordonii* (*S. gordonii*) and mediates the horizontal transfer of antibiotic resistance genes [23]. DNA released as a result of the application of H_2_O_2_ may contain antibiotic resistance genes, abandoning the previous bacterial cell and being taken up by a competent cell. As antibiotic resistance genes represent a burden for bacterial cells, once these genes are extracellularly released, the bacterial cell will gain more fitness [24].

Among ClO_2_-evolved strains, only SAL02030, SAL02017, and ATCC 14028 did not show increased resistance to ClO_2_ (Fig. 1C). Some NaClO-evolved strains showed decreased resistance to NaClO (Fig. 1D). NaClO and ClO_2_ are chlorine disinfectants with oxidizing effects. Although they are oxidizing agents such as H_2_O_2_, their efficacy and molecular mechanisms may be different. ClO_2_ has a lower redox potential than H_2_O_2_; as a result, H_2_O_2_ has shown higher oxidation of amino acids than ClO_2_. Although it has a lower redox potential, ClO_2_ has shown a higher protein denaturation ability than H_2_O_2_ [22]. NaClO is a widely used and accessible disinfectant agent. It has been used for disinfection of equipment, surfaces, laundry, and drinking water [25]. ClO_2_ has been used for municipal and hospital wastewater treatment [26, 27]. Evidence suggests that chlorine disinfection increases the abundance of antibiotic resistance genes in wastewater treatment and facilitates horizontal gene transfer between different bacterial species [28, 29]. Moreover, microbial resistance to chlorine-based disinfectants has been described [25]. This is particularly a matter of concern since most water treatment processes rely heavily on chlorine-based disinfectants.

Although ethanol has been reported to induce direct protection against subsequent lethal concentrations of ethanol in *Salmonella* [30], this was not the case in this study. Our results showed that ethanol-evolved strains did not show increased resistance to ethanol (Fig. 1E). The main reason may be that the adaptation method in our study was different from that in previous studies [30]. Ethanol resistance has been associated with the upregulation of proteins associated with purine metabolism in *Salmonella enterica* Enteritidis (*S.* Enteritidis) [31]. In *Vibrio parahaemolyticus* (*V. parahaemolyticus*), ethanol treatment may change the fatty acid profile and decrease the ratio of saturated fatty acids to unsaturated fatty acids [32].

### Cross-resistance to antibiotics emerged after the evolution of *S*. Typhimurium in disinfectants

Following evolution in the presence of different disinfectants, evolved *S*. Typhimurium strains were screened to determine whether their susceptibility changed toward a group of 14 clinically used antibiotics belonging to 9 antibiotic classes (Table 2, Additional file 2). Laboratory evolutionary experiment of *S*. Typhimurium in disinfectants selected for strains with cross-resistance to clinically relevant antibiotics in 22.9% of the evolved strains (31 out of 135) (Table 4). After exposure to H_2_O_2_, BAC, ClO_2_, NaClO, and ethanol, several strains increased their phenotypic resistance from susceptible to intermediate or resistant. Other strains also decreased their phenotypic resistance from resistant to intermediate or susceptible and from intermediate to susceptible.

**Table 4:** Antibiotic resistance profiles of evolved strains that showed MIC changes after exposure to disinfectants

Strain		Phenotypic resistance													
		Β-lactam		Aminoglycoside			Tetracycline	Macrolide	Quinolone		Phenicol	Folate inhibitor	Cephem		Carbapenem
Multidrug-resistant	ST	AMP	AMC (2:1)	GEN	KAN	STR	TET	AZI	NAL	CIP	CHL	TST (1:19)	CF	CX	IMI
SAL02010	34	R	R	S	S	R	R	S	R	I	S	R	S	S	S
Exposed to H_2_O_2_		R	R	S	S	R	R	S	R	I	S	R	S	S	S
Exposed to BAC		R	R	S	S	R	R	S	R	I	S	R	S	S	S
Exposed to ClO2		R	R	S	S	R	R	S	R	I	S	R	S	S	S
Exposed to NaClO		R	R	S	S	R	R	S	R	I	S	R	S	S	S
Exposed to ethanol		R	**S**	S	S	**﻿I**	R	S	R	I	S	R	S	S	S
SAL02073	34	R	R	R	R	R	R	S	R	R	R	R	S	S	S
Exposed to H_2_O_2_		**S**	**S**	R	**S**	R	R	S	R	**I**	R	R	S	S	S
Exposed to BAC		R	R	R	R	R	R	S	R	R	R	R	S	S	S
Exposed to ClO2		R	R	R	R	R	R	S	R	R	R	R	S	S	S
Exposed to NaClO		R	R	R	R	R	R	S	R	R	R	R	S	S	S
Exposed to ethanol		R	R	R	R	R	R	S	R	R	R	R	S	S	S
SAL02152	34	R	R	S	R	R	R	S	R	S	R	R	S	S	S
Exposed to H_2_O_2_		R	R	S	R	R	R	S	R	S	R	R	S	S	S
Exposed to BAC		R	R	S	R	R	R	S	R	***R***	R	R	S	S	S
Exposed to ClO2		R	R	S	R	R	R	S	R	**I**	R	R	S	S	S
Exposed to NaClO		R	R	S	R	R	R	S	R	S	R	R	S	S	S
Exposed to ethanol		R	**S**	S	R	R	R	S	R	S	R	R	S	S	S
SAL02135	34	R	R	S	S	R	R	S	S	S	S	R	S	S	S
Exposed to H_2_O_2_		R	R	S	S	R	R	S	S	S	S	R	S	S	S
Exposed to BAC		R	R	S	S	R	R	S	S	S	S	R	S	*I*	S
Exposed to ClO2		R	R	S	S	R	R	S	S	S	S	R	S	S	S
Exposed to NaClO		R	R	S	S	R	R	S	S	S	S	R	S	S	S
Exposed to ethanol		R	R	S	S	R	R	S	S	S	S	R	S	S	S
SAL02304	34	R	R	R	R	R	R	S	R	R	R	R	S	S	S
Exposed to H_2_O_2_		R	R	**S**	R	R	R	S	R	R	R	R	S	S	S
Exposed to BAC		R	R	R	R	R	R	S	R	R	R	R	S	S	S
Exposed to ClO2		R	R	R	R	R	R	S	R	R	R	R	S	S	S
Exposed to NaClO		R	R	R	R	R	R	S	R	R	R	R	S	S	S
Exposed to ethanol		R	R	R	R	R	R	S	R	R	R	R	S	S	S
SAL02239	19	R	R	R	I	R	R	S	S	R	R	R	S	R	S
Exposed to H_2_O_2_		R	R	R	I	R	R	S	S	**S**	R	R	S	**S**	S
Exposed to BAC		R	R	R	I	R	R	S	S	R	R	R	S	R	S
Exposed to ClO2		R	R	R	I	R	R	S	S		**S**	R	S	R	S
Exposed to NaClO		R	R	R	I	R	R	S	S	R	R	R	S	R	S
Exposed to ethanol		R	R	R	I	R	R	S	S	R	R	R	S	R	S
**Drug-resistant**															
SAL02000	19	S	S	S	S	I	I	S	R	I	S	R	S	S	S
Exposed to H_2_O_2_		S	S	S	S	I	I	S	R	I	S	R	S	S	S
Exposed to BAC		*I*	S	S	S	I	I	S	R	***R***	*I*	R	S	*I*	S
Exposed to ClO2		S	S	S	S	I	I	S	R	I	S	R	S	S	S
Exposed to NaClO		S	S	S	S	I	I	S	R	I	S	R	S	S	S
Exposed to ethanol		S	S	S	S	I	I	S	R	I	S	R	S	S	S
SAL02003	19	S	S	S	S	R	S	S	R	I	S	S	S	S	S
Exposed to H_2_O_2_		S	S	S	S	R	S	S	R	I	S	S	S	S	S
Exposed to BAC		S	S	S	S	R	S	S	R	I	S	S	S	S	S
Exposed to ClO2		S	S	S	S	R	S	S	R	I	S	***R***	S	S	S
Exposed to NaClO		S	S	S	S	R	S	S	R	I	S	S	S	S	S
Exposed to ethanol		S	S	S	S	R	S	S	R	I	S	S	S	S	S
SAL02005	19	S	S	S	S	I	I	S	R	I	S	S	S	S	S
Exposed to H_2_O_2_		S	S	S	S	I	I	S	R	I	S	S	S	S	S
Exposed to BAC		S	S	S	S	I	I	S	R	I	S	S	S	S	S
Exposed to ClO2		S	S	S	S	I	I	S	R	I	S	***R***	S	S	S
Exposed to NaClO		S	S	S	S	I	I	S	R	I	S	S	S	S	S
Exposed to ethanol		S	S	S	S	I	I	S	R	I	S	S	S	S	S
SAL02008	36	S	S	S	S	S	S	S	S	R	S	R	S	S	S
Exposed to H_2_O_2_		S	S	S	S	*I*	S	S	S	**I**	S	**S**	S	S	S
Exposed to BAC		S	S	S	S	S	S	S	S	R	S	R	S	S	S
Exposed to ClO2		S	S	S	S	S	S	S	S	R	S	R	S	S	S
Exposed to NaClO		S	S	S	S	S	S	S	S	R	S	R	S	S	S
Exposed to ethanol		S	S	S	S	S	S	S	S	R	S	R	S	S	S
SAL02219	19	S	S	S	S	I	I	S	R	I	I	R	S	S	S
Exposed to H_2_O_2_		S	S	S	S	**S**	**S**	S	R	I	**S**	**S**	S	S	S
Exposed to BAC		***R***	S	S	S	I	I	S	R	I	I	R	S	S	S
Exposed to ClO2		S	S	S	S	I	I	S	R	I	I	R	S	S	S
Exposed to NaClO		S	S	S	S	I	**S**	S	R	I	**S**	**S**	S	S	S
Exposed to ethanol		S	S	S	S	I	**S**	S	R	I	**S**	R	S	*I*	S
SAL02030	34	S	S	S	R	R	I	S	R	I	S	S	S	S	S
Exposed to H_2_O_2_		S	S	S	R	R	I	S	R	I	S	S	S	S	S
Exposed to BAC		S	S	S	R	R	I	S	R	I	S	S	S	***R***	S
Exposed to ClO2		S	S	S	R	R	I	S	R	I	S	S	S	*I*	S
Exposed to NaClO		S	S	S	R	R	I	S	R	I	S	S	S	S	S
Exposed to ethanol		S	S	S	R	R	I	S	R	I	S	S	S	S	S
SAL02069	36	S	S	S	S	I	S	S	S	I	S	R	S	S	S
Exposed to H_2_O_2_		S	S	S	S	I	S	S	S	I	S	R	S	S	S
Exposed to BAC		S	S	S	S	I	S	S	S	I	S	R	S	*I*	S
Exposed to ClO2		S	S	S	S	I	S	S	S	I	S	R	S	S	S
Exposed to NaClO		S	S	S	S	I	S	S	***R***	***R***	S	R	S	S	S
Exposed to ethanol		S	S	S	S	**S**	S	S	S	I	S	R	S	*I*	S
SAL02203	36	S	S	S	S	S	S	S	S	R	S	S	S	S	S
Exposed to H_2_O_2_		S	S	S	S	S	S	S	S	R	S	S	S	S	S
Exposed to BAC		S	S	S	S	S	S	S	***R***	R	*I*	S	S	S	S
Exposed to ClO2		S	S	S	S	S	S	S	S	R	S	S	S	S	S
Exposed to NaClO		S	S	S	S	S	S	S	S	R	S	S	S	S	S
Exposed to ethanol		S	S	S	S	S	S	S	S	R	S	S	S	S	S
**Susceptible**															
SAL01711	34	S	S	S	S	R	S	S	S	S	S	R	S	S	S
Exposed to H_2_O_2_		S	S	S	S	R	S	S	S	S	S	R	S	S	S
Exposed to BAC		S	S	S	S	R	S	S	S	S	S	R	S	S	S
Exposed to ClO2		S	S	S	S	R	S	S	S	S	S	R	S	S	S
Exposed to NaClO		S	S	S	S	R	S	S	S	S	S	R	S	S	S
Exposed to ethanol		S	S	S	S	*I*	S	S	S	*I*	S	R	S	*I*	S
SAL02017	19	S	S	S	S	S	S	S	S	S	S	S	S	S	S
Exposed to H_2_O_2_		S	S	S	S	S	S	S	S	S	S	S	S	S	S
Exposed to BAC		S	S	S	S	S	S	S	S	S	S	S	S	S	S
Exposed to ClO2		S	S	S	S	S	S	S	S	***R***	S	***R***	S	S	S
Exposed to NaClO		S	S	S	S	S	S	S	S	S	S	S	S	S	S
Exposed to ethanol		S	S	S	S	S	S	S	S	S	S	S	S	S	S
SAL02070	34	S	S	S	S	I	S	S	S	S	S	R	S	S	S
Exposed to H_2_O_2_		S	S	S	S	I	S	S	S	S	S	R	S	S	S
Exposed to BAC		S	S	S	S	***R***	S	S	S	S	S	R	S	*I*	S
Exposed to ClO2		S	S	S	S	I	S	S	S	*I*	S	**S**	S	S	S
Exposed to NaClO		S	S	S	S	I	S	S	S	S	S	R	S	S	S
Exposed to ethanol		S	S	S	S	***R***	S	S	S	S	S	R	S	*I*	S
SAL02685	99	S	S	S	S	I	S	S	S	S	S	R	S	S	S
Exposed to H_2_O_2_		S	S	S	S	I	S	S	S	S	S	R	S	S	S
Exposed to BAC		S	S	S	S	I	S	S	S	S	S	R	S	S	S
Exposed to ClO2		S	S	S	S	I	S	S	S	S	S	R	S	S	S
Exposed to NaClO		S	S	S	S	***R***	S	S	***R***	S	S	R	S	S	S
Exposed to ethanol		S	S	S	S	I	S	S	S	S	S	R	S	S	S
*Reference*															
ATCC 14028	19	S	S	S	S	R	S	S	S	S	S	S	S	S	S
Exposed to H_2_O_2_		S	S	S	S	R	S	S	S	*I*	S	S	S	S	S
Exposed to BAC		S	S	S	S	R	S	S	S	*I*	S	S	S	S	S
Exposed to ClO2		S	S	S	S	R	S	S	S	*I*	S	***R***	S	S	S
Exposed to NaClO		S	S	S	S	R	S	S	S	S	S	S	S	S	S
Exposed to ethanol		S	S	S	S	R	S	S	S	S	S	S	S	S	S

R resistant, I intermediate, S susceptible

Although the results varied, various BAC-evolved strains showed a tendency of increased resistance to quinolone and cephem antibiotics, and various ClO_2_-evolved strains showed increased resistance to TST. In *Listeria monocytogenes* (*L. monocytogenes*), exposure to increasing concentrations of BAC has been shown to select for resistance to various antibiotics, including cefotaxime, cephalothin, and CIP, and has been suggested to be efflux pump-mediated [33]. Exposure to BAC and chlorine-based disinfectants enhances cross-resistance to antibiotics in Gram-negative bacteria such as *Salmonella* [34]. Zeng et al. [35] found that low to medium BAC exposure in the soil microcosms in agricultural soils selected for an increased number of antibiotic resistance genes. As BAC is used in many consumer products, it inevitably ends up in surface water used for drinking water treatment. Laboratory-scale microcosm experiments have shown that BAC at a wide concentration range (0.1–500 μg/L) selects for resistance to ciprofloxacin and sulfamethoxazole and increases the abundance of the antibiotic resistance genes *sul1* and *bla*_TEM_ [36]. This evidence is particularly concerning because BAC may enter drinking water treatment plants and potentially expose people to antibiotic resistance from drinking water. Chlorine-based disinfectants have been shown to promote disinfectant and antibiotic resistance in Pseudomonas spp. through the SOS response triggered by oxidative stress [37]. Moreover, chlorine disinfection to drinking water promotes the exchange of antibiotic resistance genes between bacterial cells [29].

In the current literature, no cross-resistance to antibiotics has been described after low-level exposure to H_2_O_2_ and ethanol. This explains why in this study, only one strain (SAL02008) showed cross-resistance to STR and CIP, and three strains showed decreased resistance after exposure to H_2_O_2._ These strains included SAL02073, which showed decreased resistance to AMP, AMC, KAN, and CIP; SAL02304, which showed decreased resistance to GEN; SAL02239 which showed decreased resistance to CIP and CX; and SAL02219, which showed decreased resistance to STR, TET, CHL, and TST. The number of strains that showed increased resistance to antibiotics was also the same as the number of strains that showed decreased resistance after exposure to ethanol [34]. In a previous study, H_2_O_2_ treatment enhanced the removal of antibiotic resistance genes from sediments [38]. H_2_O_2_ has the ability to enhance the release of DNA from bacterial cells [23].

**Fig. 2:**
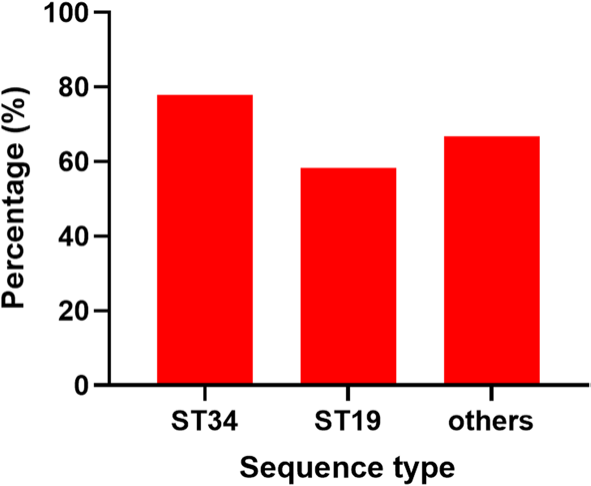
Percentages of ST34, ST19, and other STs strains that showed changes in the antibiotic resistance profile. Other STs include ST36, ST99, and ST313

**Fig. 3:**
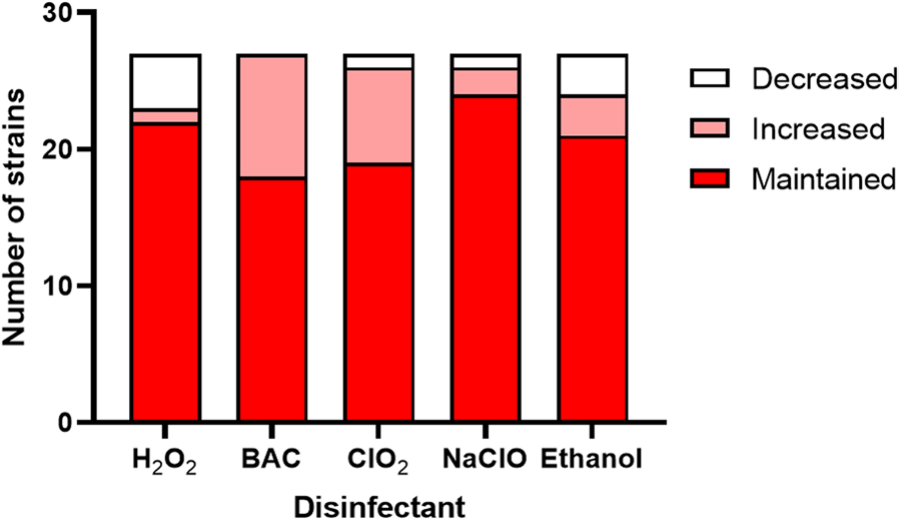
Number of strains that decreased, increased, or maintained resistance to a group of 14 clinically important antibiotics after exposure to increasing concentrations of H_2_O_2_, BAC, ClO_2_, NaClO, or ethanol

MLST patterns showed the classification of five STs, including ST34, ST19, ST36, ST99, and ST313. Multilocus STs ST34 and ST19 of *S*. Typhimurium have been classified as the major STs causing human salmonellosis worldwide and have been reported in several cases of human infections [5]. The other STs, including ST36, ST99, and ST313, were classified as minor STs and grouped together. As shown in Fig. 2, 77.78% of ST34 and 58.33% of ST19 strains showed changes in their antibiotic resistance profiles following exposure to disinfectants compared to 66.67% of other STs. Recent findings highlight the increasing prevalence of multidrug-resistant strains of the monophasic variant of *S*. Typhimurium ST34, which has been isolated from food, human, farm animal, and environmental sources [3]. Luo et al. [39] found that *S*. Typhimurium ST34 was the predominant lineage carrying the mobilized colistin resistance gene *mcr,* accounting for 30.12% of all *mcr*-positive isolates (Fig. 3). ST34 is often regarded as a pandemic lineage and global menace given its multidrug resistance and rapid dissemination at the global scale [40]. It has outcompeted the traditional ST19 lineage, exhibiting increasing prevalence in recent years [39].

### Different disinfectants selected for different evolutionary traits

Strains exposed to different disinfectants showed different evolutionary traits. The results showed that antibiotic resistance decreased in four out of 27 strains following H_2_O_2_ exposure (Fig. 3). Strains SAL02073, SAL02239, and SAL02219 decreased their resistance to several antibiotics, including AMP, AMC, KAN, CIP, CX, STR, TET, CHL and TST, while SAL02304 decreased resistance to GEN. H_2_O_2_ is a strong oxidizing agent [41], and studies reporting cross-resistance to antibiotics following exposure to hydrogen peroxide have not been reported or are limited [34]. Previous studies have shown that hydrogen peroxide is effective for removing antibiotic resistance genes [38].

The highest number of strains that showed increased resistance to antibiotics was obtained after exposure to BAC. A total of nine out of 27 strains showed increased resistance to at least one antibiotic, including CIP, NAL, STR, CX, AMP, and CHL, following exposure to BAC. Previous studies have reported that BAC increased the antibiotic resistance of *A. baumannii*, *P. aeruginosa*, and *E. coli* [9–11]. The mechanisms for increased antibiotic resistance include the overexpression of multidrug efflux pump genes [9]. Repeated exposure of *S.* Typhimurium to quaternary ammonium compounds may select for increased resistance to several antibiotics, including CHL, TET, AMP, acriflavine, and triclosan, mediated by overexpression of the AcrAB efflux pump [42].

Following exposure to ClO_2_, eight strains increased resistance to at least one antibiotic, including CIP, TST, and CX, and one (SAL02239) decreased resistance to CHL. The exact mechanism of action of ClO_2_ remains unclear, with some studies suggesting DNA damage and others suggesting protein denaturation by oxidation [43]. Previous studies have suggested that preexposure of *S.* Typhimurium to ClO_2_ increased resistance to STR, erythromycin, rifampicin, and CHL [44]. NaClO was the disinfectant that had the lowest effect on antibiotic resistance. Two strains (SAL02069 and SAL02685) showed increased resistance to NAL, CIP, and STR, and one strain (SAL02219) showed decreased resistance to STR, TET, CHL, and TST following exposure to NaClO. The reason for this is probably due to neutralization of NaClO by the organic load in the culture medium [25], which avoids selective pressure on bacterial cells. Previous studies have shown that some strains of *Salmonella* species have developed resistance to antibiotics, including GEN, CX, and NAL, after adaptation to NaClO [45].

Chlorine-based disinfectants are oxidizing agents. Oxidative stress induced by treatment with ClO_2_ and NaClO causes gene overexpression and therefore results in an increased antibiotic resistance phenotype [37]. Chlorine-based disinfectants also mediate the horizontal gene transfer of resistance genes [46]. Because selection is not uniform and most of the time depends on the probability of the sublethal concentration and the starting genetic background [21], susceptible populations may sometimes outcompete resistant populations and display a phenotype with decreased resistance.

After ethanol exposure, three strains (SAL02010, SAL02152, and SAL02219) decreased their antibiotic resistance to some antibiotics, including AMC, STR, TET, and CHL, while the other three strains (SAL02069, SAL01711, and SAL02070) increased their resistance to at least one antibiotic, including CX, STR, and CIP. However, the strains did not exhibit increased resistance to ethanol. Exposure to ethanol may affect the expression of sigma factor-regulated genes that contribute to the biogenesis of cell membranes, lipid transport and metabolism, and oxidative stress resistance [47]; hence, the different outcomes in antibiotic resistance change.

### Mutations in evolved strains correlate with antibiotic cross-resistance

**Fig. 4:**
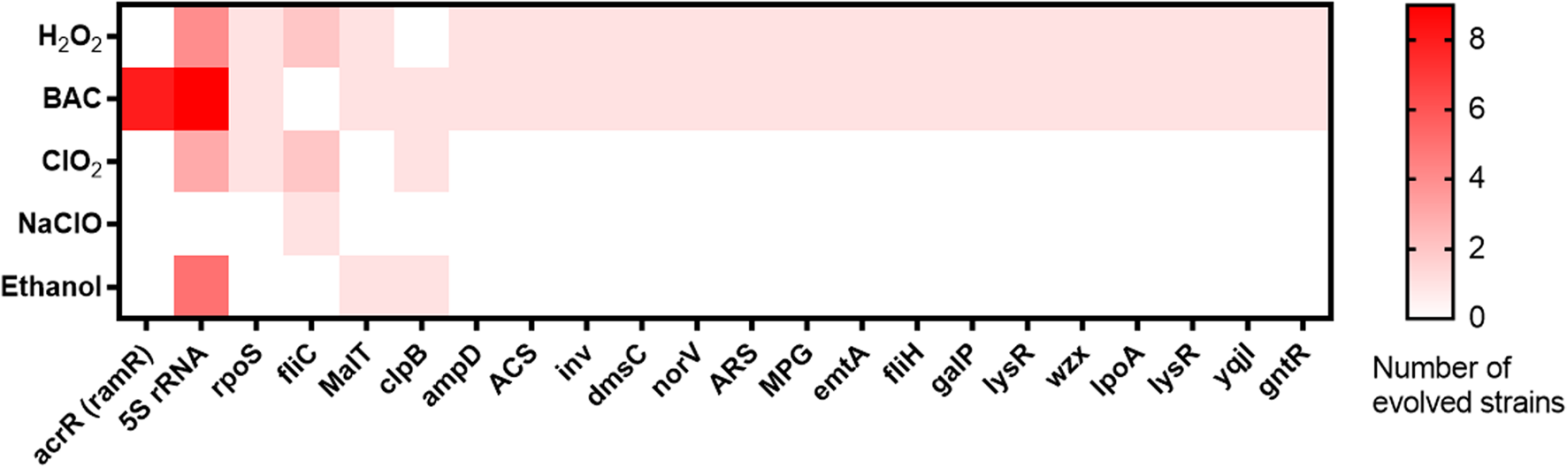
Mutations found in the evolved strains following exposure to five disinfectants. Only genes mutated in two or more of the 35 evolved strains are shown in this heatmap. The color code indicates the number of cases when the same gene was mutated in different strains that evolved under the same disinfectant

For the obtained *S*. Typhimurium evolved strains, some of the mutated genes have been previously linked to antibiotic resistance. These genes included those that encode RNA polymerase sigma factors, chaperone proteins, transcriptional regulators of multidrug efflux pumps, multidrug efflux pumps, transporters, and rRNA subunits (Fig. 4). A more detailed and complete description of the mutations identified can be found in Additional file 3.

In this study, 18 out of 31 evolved strains showed mutations in different sites of the 5S rRNA gene. Bacteria have 70S ribosomes that are composed of two subunits. The large 50S subunit includes the 23S and 5S rRNAs, while the small 30S subunit includes the 16S rRNA [48]. Mutations in the rRNA gene have been linked with acquired resistance to antibiotics [49, 50].

After evolution in ClO_2_, strain SAL02017 had an N98H point mutation in the *rpoS* gene. Mutations in the stationary phage sigma factor *rpoS* have been implicated in enhanced resistance to antibiotics in *P. aeruginosa* PAO1 [51]. In *E. coli*, *rpoS* mutations promote an extracellular factor that increases the production of biofilm during exponential phase [52].

Eight out of nine strains exposed to BAC that showed increased resistance to antibiotics also showed mutations in different sites of the acrR (*ramR)* gene including T18P, G42E, A37V, G25A, Δ7 bp, and Δ46 bp, suggesting that BAC may select for mutations in the *ramR* gene and increase antibiotic resistance. Mutations in the *ramR* gene, which is a local repressor of *ramA*, have been associated with increased resistance to antibiotics in *Salmonella*. Mutations in *ramR* resulted in a fourfold increase in the expression of *ramA* and AcrAB efflux pump, which resulted in a fourfold increase in MIC of fluoroquinolones, phenicols, and tetracycline antibiotics [53].

A substitution of asparagine for an alkaline amino acid lysine point mutation (N40K) of *yqjA* in strain SAL02030 after BAC evolution was observed. This may be the reason for the decreased susceptibility to CX, a weakly acidic antibiotic. The N40K mutation may increase the ability of YqjA to expel the acidic antibiotic CX. YqjA is a membrane transporter that belongs to the DedA family of membrane proteins present in bacteria and possesses an antibiotic resistance function. Moreover, Yqja is directly involved in drug efflux [54]. In *Enterobacteriaceae*, it functions as a membrane cation/proton antiporter important for pH homeostasis. It has been reported that substitutions of acidic amino acids at positions E39 and D15 for amidic amino acids impaired the ability of *E. coli* to survive alkaline pH [55].

Not all mutated genes in the evolved strains can be correlated with antibiotic resistance based on the current data available in the literature. Previous studies have associated mutations induced by biocides in enzymes and membrane maintenance genes with cross-resistance to antibiotics in *E. coli* [56]. Chaperones such as the DnaK system have also been associated with antibiotic resistance in *Mycobacterium tuberculosis* by associating with many drug targets, including RNA polymerase and the ribosome [57]. ClpB is an ATP-dependent unfoldase chaperone protein that is able to disaggregate stress-denatured bacterial proteins and plays an important role in survival under different stresses [58], so it works as a mechanism of stress resistance.

Peptidyl‑prolyl cis‑trans isomerase PpiD (EC 5.2.1.8) has been found to be fundamental for the folding of outer membrane proteins and can be transcribed by the two-component system CpxR-CpxA in *E. coli*. CpxR-CpxA is involved in the function of multidrug efflux pumps in *E. coli* [59]. We observed a mutation in the coding region of the PpiD gene in strain SAL02152 after ClO_2_ exposure, which we hypothesized was the main driver of increased resistance.

The oxidoreductase YeaE is a member of the aldo–keto reductase superfamily. This enzyme catalyzes redox transformations in biosynthesis, intermediary metabolism, and detoxification. It has a role in drug metabolism and detoxification [60].

Class 1b ribonucleoside‑diphosphate reductase subunit beta catalyzes the synthesis of all four deoxyribonucleotides for DNA synthesis by reducing ribonucleotides. This synthesis is transcriptionally regulated in most organisms [61]. A point mutation of P147Q in class 1b ribonucleoside‑diphosphate reductase subunit beta was found in strain D23580 after exposure to ClO_2_.

### Relative fitness of evolved strains

**Fig. 5:**
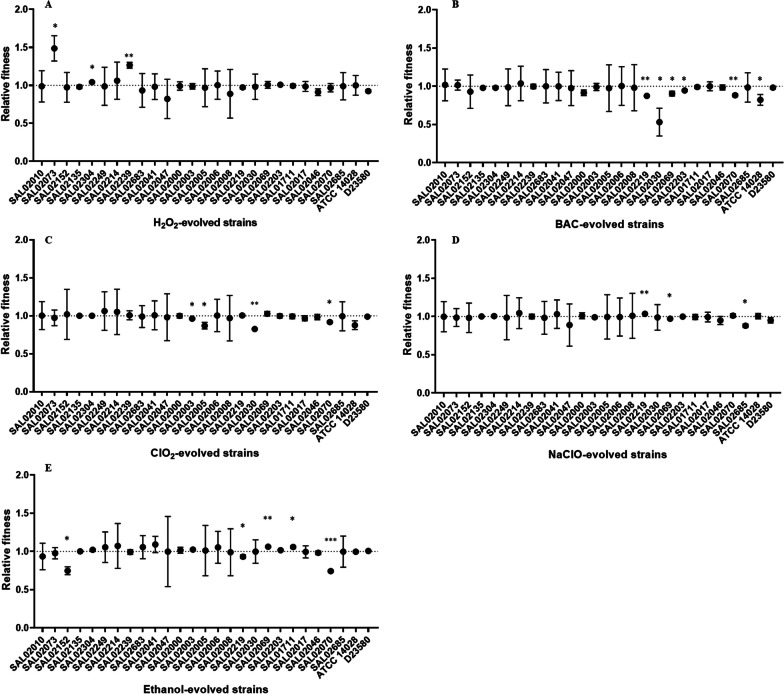
Relative fitness of strains evolved in H_2_O_2_ (**A**), BAC (**B**), ClO_2_ (**C**), NaClO (**D**), and ethanol (**E**) in antimicrobial-free medium. Growth curves of the evolved strains before and after exposure to disinfectants were recorded in disinfectant-free LB broth. The fitness of the strain was measured as the area under the growth curve. The relative fitness of the evolved strains was calculated by dividing the fitness of evolved strains by the fitness of the corresponding wild-type strain. Significance of difference of evolved to wild type strain indicated by asterisks: ^*^P ≤ 0.05; ^**^P ≤ 0.01 (one sample *t* test for μ = 1)

The fitness of each strain was measured by calculating the area under the growth curve in a disinfectant-free medium. The fitness values were then used to calculate the relative fitness of each evolved strain with respect to their corresponding parental strain. It was found that evolved strains with increased MICs of antibiotics showed decreased relative fitness. On the other hand, evolved strains that showed decreased MICs to antibiotics showed increased relative fitness (Fig. 5).

Following exposure to H_2_O_2_, strains SAL02073, SAL02304, and SAL02239 showed significant (*P* ≤ 0.05) increases in relative fitness (Fig. 5A). An antibiotic MIC decrease was associated with fitness gain, as reported in previous studies [21]. These H_2_O_2_-evolved strains showed reversion of resistance to antibiotics, changing from resistant to susceptible and intermediate. SAL02073 changed from resistant to susceptible to AMP, AMC, and KAN and from resistant to intermediate to CIP. SAL02304 changed from resistant to susceptible to GEN. SAL02239 changed from resistant to susceptible to CIP and CX (Table 4).

Several BAC-evolved strains showed a significant (*P* ≤ 0.05) decrease in relative fitness (Fig. 5B). Strains SAL02219, SAL02030, SAL02069, SAL02203, SAL02070, and ATCC 14252 showed decreased relative fitness after exposure to BAC. These evolved strains also showed increased resistance to at least one antibiotic (Table 4). The wild types of these strains were classified as drug-resistant and drug-susceptible. Interestingly, strains that were classified as multidrug resistant and that showed increased MICs to antibiotics after exposure to BAC did not show a significant decrease in relative fitness. This is probably due to the burden they already carry for the multidrug resistance phenotype, which affects their fitness. The addition of a more resistant phenotype did not have a significant effect on their already low fitness.

Four ClO_2_-evolved strains showed a significant (*P* ≤ 0.05) decrease in relative fitness, including SAL02003, SAL02005, SAL02030, and SAL02070 (Fig. 5C). These four evolved strains also showed increased MICs of antibiotics. The evolved strain SAL02152, which showed increased MIC to CIP after ClO_2_ exposure, did not show decreased relative fitness, similar to the results of BAC-evolved strains.

The three NaClO-evolved strains showed significant (*P* ≤ 0.05) differences in relative fitness (Fig. 5D). SAL02219, which showed a decreased MIC to TET, CHL, and TST, showed a significant (*P* ≤ 0.05) increase in relative fitness. On the other hand, SAL02069 and SAL02685, which showed increased MICs to antibiotics, showed significantly (*P* ≤ 0.05) decreased relative fitness. Chlorine-based disinfectants such as ClO_2_ and NaClO are oxidizing agents with a mechanism of one-electron transfer that attacks the electron-rich core in proteins and enzymes, therefore giving them broad-spectrum antimicrobial activity [62]. The oxidative stress induced by ClO_2_ and NaClO causes antibiotic resistance gene overexpression and therefore affects the fitness of the bacterial cell [37].

Significant differences (*P* ≤ 0.05) in relative fitness were found in ethanol-evolved strains (Fig. 5E). SAL02152 after ethanol exposure showed a significant (*P* ≤ 0.05) decrease in relative fitness. However, it showed decreased MIC to AMC. SAL02219 and SAL02069 also showed significant (*P* ≤ 0.05) differences in relative fitness. In previous studies, ethanol has been shown to induce cross-protection against freezing stress in *S*. Enteritidis [30]. However, no evidence of cross-resistance to antibiotics following exposure to ethanol has been described. Ethanol changes the fatty acid profile of the cell membrane and regulatory pathways associated with metabolism [31, 32]. Hence, there are different outcomes in relative fitness.

In evolutionary biology, fitness is an important characteristic [63]. The evolutionary adaptation of bacteria to a stressful environment may decrease or increase fitness in other environments [64]. Because mutations that confer antibiotic resistance target important biological functions in the cell, they may be expected to provide a fitness cost [65]. Resistance plasmids can also impose fitness costs in bacterial cells in the absence of antibiotics [66]. In antibiotic-free environments, resistant bacteria suffer a fitness cost (i.e., reduced growth rate) and are eventually outcompeted by their susceptible and more fit counterparts [67]. The evolution of reduced susceptibility to antimicrobials is often accompanied by associated costs, especially when the change is mutational, resulting in resistance. For instance, efflux pumps consume cell energy and indiscriminately remove some useful metabolic substances from the bacterial cell. When a disinfectant selects for a strain that overexpresses efflux, this strain will pay the costs of efflux regardless of the presence of the disinfectant [68]. However, selection is not uniformly costly, and extended exposure may select for compensatory mutations or genetic reversion of the original mutations [21]. Variations in the costs of resistance can emerge because some mutations may or may not be costly [65]. These results suggest that the mutations resulting from exposure to disinfectants specifically BAC, ethanol, and chlorine-based disinfectants, are the main source of fitness cost.

Increased resistance of strains to antimicrobials makes such strains a potential risk, although there will be a fitness cost in the absence of selective pressure. Evidence shows that the fitness costs of carrying antibiotic resistance in resistant bacteria will allow susceptible bacteria to outcompete antibiotic-resistant bacteria in the absence of selective pressure from antimicrobials [69]. These results clearly demonstrate that the evolution of resistance to antibiotics is accompanied by a fitness cost and that reversion of resistance improves the fitness of the bacterial cell. Changes in disinfectant resistance did not significantly affect the fitness of evolved strains. Fitness loss and fitness gain were associated with the phenotype of antibiotic resistance but not disinfectant resistance.

## Conclusions

This study shows evidence that disinfectants may promote the emergence of antibiotic resistance in bacteria and that different disinfectants may force evolution in different directions. The frequency at which *S*. Typhimurium strains evolve may be mediated by the MLST of each strain. This study also highlights the need for a more responsible use of disinfectants to avoid long-term exposure to disinfectants and potential selective pressure for cross-resistant strains, especially those belonging to the ST34 lineage, which is often regarded as a pandemic lineage. Further studies should emphasize the mechanisms of each disinfectant on cross-resistance to antibiotics. Potential consequences for the host should also be explored in future studies.

### Supplementary Information

**Additional file 1**. Bacterial strains and culture conditions.

**Additional file 2**. Cross-resistance to antibiotics emerged after the evolution of S. Typhimurium in disinfectants.

**Additional file 3**. Mutations in evolved strains correlate with antibiotic cross-resistance.

## Data Availability

The datasets used and/or analyzed during the current study are available from the corresponding author on reasonable request.
